# Balance Right in Multiple Sclerosis (BRiMS): a feasibility randomised controlled trial of a falls prevention programme

**DOI:** 10.1186/s40814-020-00732-9

**Published:** 2021-01-04

**Authors:** H. Gunn, K. N. Stevens, S. Creanor, J. Andrade, L. Paul, L. Miller, C. Green, P. Ewings, A. Barton, M. Berrow, J. Vickery, B. Marshall, J. Zajicek, J. A. Freeman

**Affiliations:** 1 Faculty of Health, School of Health Professions, Peninsula Allied Health Centre, University of Plymouth, Derriford Road, Plymouth, PL6 8BH England; 2 Faculty of Health, Medical Statistics Group, Room N15, Plymouth Science Park, Plymouth, PL6 8BX England; 3 Peninsula Clinical Trials Unit, University of Plymouth, Room N16, Plymouth Science Park, Plymouth, PL6 8BX England; 4 University of Exeter Medical School, College of Medicine & Health, University of Exeter, Exeter, England; 5 Faculty of Health, School of Psychology, University of Plymouth, Portland Square Building, Drake Circus Campus, Plymouth, PL4 8AA England; 6 School of Health & Life Sciences, Glasgow Caledonian University, Cowcaddens Road, Glasgow, G4 0BA Scotland; 7 Douglas Grant Rehabilitation Unit, Ayrshire Central Hospital, Kilwinning Road, Irvine, KA12 8SS Scotland; 8 University of Exeter Medical School, Health Economics Group, University of Exeter, St. Luke’s Campus, Exeter, EX1 2LU England; 9 NIHR Research Design Service (South West), Musgrove Park Hospital, Taunton, TA1 5DA England; 10 Faculty of Medicine and Dentistry, NIHR Research Design Service South West, ITTC Building, Plymouth Science Park, Plymouth, PL6 8BX England; 11 School of Medicine, Medical and Biological Sciences, University of St. Andrews, North Haugh, St. Andrews, KY16 9TF Scotland

**Keywords:** Secondary progressive multiple sclerosis, Exercise, Self-management, Mobility, Accidental falls, Balance, Quality of life, Feasibility randomised controlled trial

## Abstract

**Background:**

Balance, mobility impairments and falls are problematic for people with multiple sclerosis (MS). The “Balance Right in MS (BRiMS)” intervention, a 13-week home and group-based exercise and education programme, aims to improve balance and minimise falls. This study aimed to evaluate the feasibility of undertaking a multi-centre randomised controlled trial and to collect the necessary data to design a definitive trial.

**Methods:**

This randomised controlled feasibility study recruited from four United Kingdom NHS clinical neurology services. Patients ≥ 18 years with secondary progressive MS (Expanded Disability Status Scale 4 to 7) reporting more than two falls in the preceding 6 months were recruited. Participants were block-randomised to either a manualised 13-week education and exercise programme (BRiMS) plus usual care, or usual care alone.

Feasibility assessment evaluated recruitment and retention rates, adherence to group assignment and data completeness. Proposed outcomes for the definitive trial (including impact of MS, mobility, quality of life and falls) and economic data were collected at baseline, 13 and 27 weeks, and participants completed daily paper falls diaries.

**Results:**

Fifty-six participants (mean age 59.7 years, 66% female, median EDSS 6.0) were recruited in 5 months; 30 randomised to the intervention group. Ten (18%) participants withdrew, 7 from the intervention group. Two additional participants were lost to follow up at the final assessment point. Completion rates were > 98% for all outcomes apart from the falls diary (return rate 62%).

After adjusting for baseline score, mean intervention—usual care between-group differences for the potential primary outcomes at week 27 were MS Walking Scale-12v2: − 7.7 (95% confidence interval [CI] − 17.2 to 1.8) and MS Impact Scale-29v2: physical 0.6 (CI − 7.8 to 9), psychological − 0.4 (CI − 9.9 to 9). In total, 715 falls were reported, rate ratio (intervention:usual care) for falls 0.81 (0.41 to 2.26) and injurious falls 0.44 (0.41 to 2.23).

**Conclusions:**

Procedures were practical, and retention, programme engagement and outcome completion rates satisfied a priori progression criteria. Challenges were experienced in completion and return of daily falls diaries. Refinement of methods for reporting falls is therefore required, but we consider a full trial to be feasible.

**Trial registration:**

ISRCTN13587999

Date of registration: 29 September 2016

## Key messages on feasibility

(i) What uncertainties existed regarding feasibility? It was unknown whether the recruitment strategies and processes would be effective, and whether participants would be able to maintain engagement with the trial and the BRiMS programme. There were also uncertainties about the choice of a primary outcome for a full effectiveness trial, and the methods used for collecting prospective falls data.
(ii) What are the key findings on feasibility from this study? The trial methods were feasible and effective in recruiting and retaining participants, although some changes to the BRiMS programme were indicated to reduce attrition. Most outcome measures had satisfactory completion; however, there were challenges in the methods of collecting falls data.
(iii) What are the implications of the feasibility findings on the design of the main study? The findings indicate that a large-scale trial is feasible; however, refinement of falls reporting methods and development of BRiMS programme delivery methods are recommended prior to progressing further.

## Background

Multiple sclerosis (MS) is an incurable, unpredictable but typically progressive, life-long, neurological condition, affecting approximately 100,000 people in the UK (UK) [1]. It is the most common cause of neurological disability in young adults. Although most people start with a relapsing-remitting (RR) disease course, approximately two-thirds move to a progressive phase, with a steady rise in the proportion of progressive cases as the disease advances.

Within approximately 15 years of diagnosis, an estimated 50% of people are unable to walk unaided, and eventually 25% are dependent on a wheelchair [2]. An important contributor to this is impaired balance, which is reported by approximately 75% of people with MS [3]. Mobility is more compromised in those with secondary progressive MS (SPMS) compared to RR MS [4]. Our previous work suggests that falls may be an early marker of mobility deterioration associated with disease progression [5]. Rehabilitation interventions which improve balance and mobility, and therefore decrease the risk of falls, may slow this deterioration, providing a persuasive argument for ensuring this should be a clinical priority. With only limited medical interventions available for this patient group, rehabilitation programmes are considered key to management but currently lack a robust evidence base [6].

In partnership with service users, providers of rehabilitation services, other key stakeholders (including service commissioners) and international collaborators, our ongoing research programme has systematically developed ‘Balance Right in MS’ (BRiMS), an innovative 13-week evidence-based, user-focused, manualised, self-management programme, designed to improve safe mobility and reduce falls for people with MS [5, 7, 8]. The programme includes personalised education and exercise and motivation components, designed to address modifiable fall risk factors, and enable self-management by use of mobility, safety and falls risk management strategies.

## Aim

The overall aim of this study was to evaluate the feasibility of undertaking a multi-centre randomised controlled trial (RCT) to compare BRiMS plus usual care with usual care alone, and to collect the necessary data to design a definitive trial. The study objectives were to determine:

1. Feasibility:
  Suitability, utility and acceptability of the study procedures
  Appropriateness of eligibility criteria,
  Viability of recruitment and randomisation procedures,
  Retention rates,
  Participant engagement throughout the study,
  Adverse events.
2. Potential definitive trial outcomes:
  The selection of primary and secondary outcome measures including:
  Their characteristics and rates of completion i.e. baseline scores, distributional properties standard deviations,
  Responsiveness and to help determine the sample size for the RCT.
3. Health economics objectives:
  Estimates of resource use and related costs associated with delivery of the BRiMS intervention

## Methods

The study was undertaken according to the methods detailed in our protocol [9], which are briefly summarised below.

### Study design

This was a pragmatic, mixed-methods, multi-centre, feasibility, individually randomised, group treatment RCT, with blinded outcome assessment and embedded process evaluation.

### Participants

The target population was English-speaking men and women, aged ≥ 18 years, with a confirmed diagnosis of SPMS (Expanded Disability Status Scale (EDSS) 4-7 inclusive), who reported having walking difficulties and more than two falls in last 6 months. People were excluded if they

- had ever had previous treatment with alemtuzumab;
- were within 6 months of ceasing natalizumab; or within 3 months of ceasing any other MS disease-modifying drug;
- reported a relapse within the last month as defined: ﻿“the appearance of new symptoms, or the return of old symptoms, for a period of 24 h or more—in the absence of a change in core body temperature or infection”) [10];
- had been referred to a falls management programme within the previous 6 months, or
- were participating in a concurrent trial.

### Recruitment

The study recruited from four UK NHS clinical neurology services. Potential participants were identified through local and national advertising, adoption on to the local NIHR Clinical Research Network portfolio and via the caseload of local MS clinicians. Due to the nature of the group-based intervention and to facilitate randomisation, participants were recruited in blocks of 8–12 individuals (for full details, refer to the study protocol [9]).

### Study procedures

**Fig. 1:**
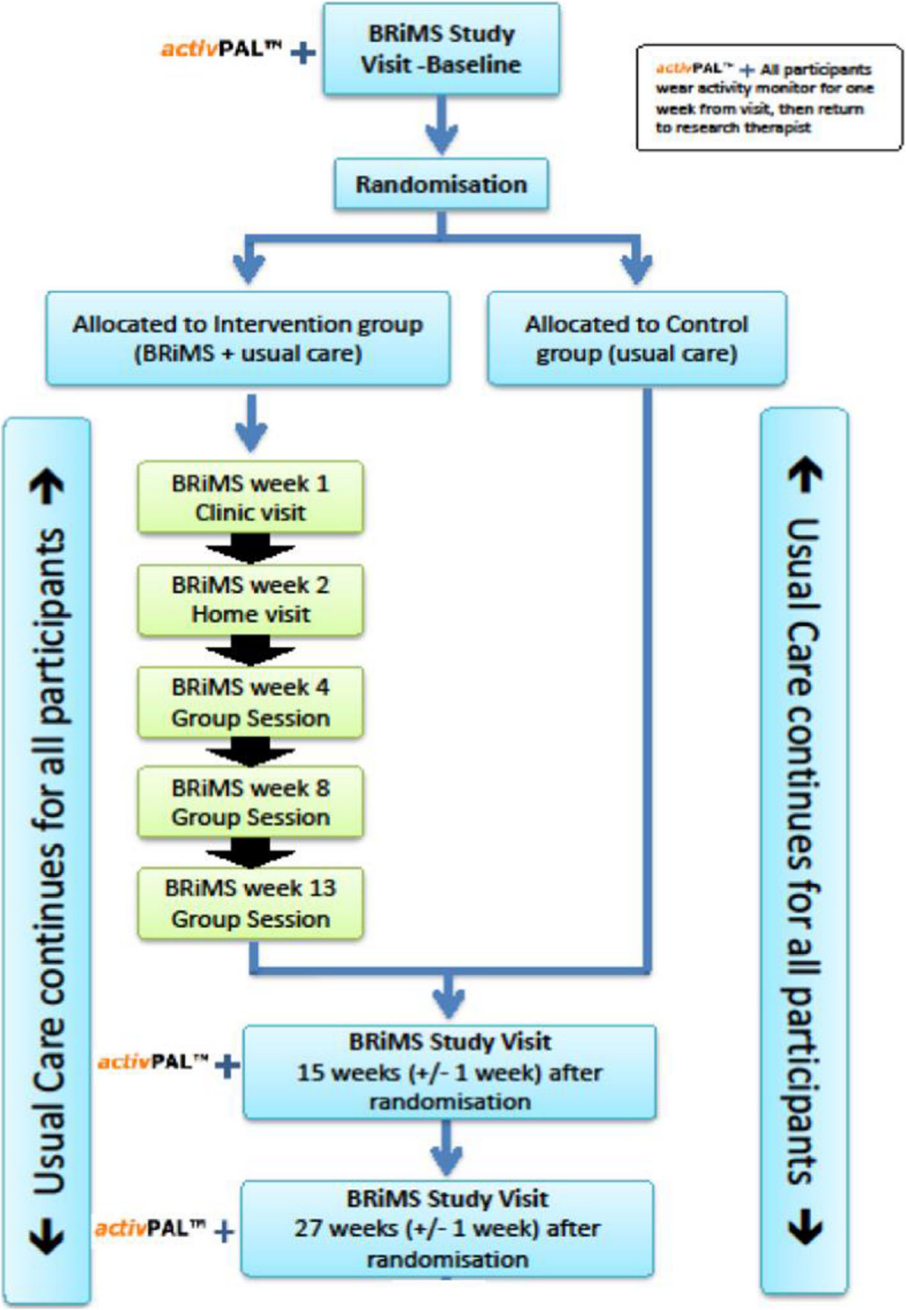
Participant pathway

The participant pathway is detailed in Fig. 1. Site-based research therapists screened potential participants by telephone interview. Final eligibility checking, informed consent and baseline measures were undertaken at a single face-to-face meeting at a local healthcare venue, no more than 2 weeks prior to the pre-scheduled randomisation date for each BRiMS delivery. Randomisation was undertaken via a secure web-based system by staff from the UK Clinical Research Collaboration registered Peninsula Clinical Trials Unit who were not involved in the delivery of the study. Participants were randomised in block sizes of 8–12. Within each block, participants were individually randomised to BRiMS plus usual care (BRiMS) or usual care only on a 1:1 basis and informed of this allocation by email.

Participants were followed-up on two occasions: at 13 weeks (± 1 week) and 27-weeks (± 1 week) following randomisation.

### Staffing

BRiMS sessions were delivered by trained treating physiotherapists [11]. Research assessments were undertaken by research physiotherapists who were aware of the study aims but were blinded to individuals’ allocated group.

### Interventions (see Fig. 2)

In addition to usual care, participants allocated to the BRiMS programme were asked to undertake a home exercise and falls prevention education programme. This aimed to support participants to achieve a minimum of 120 min of individualised, progressive, gait, balance and functional training per week, and to complete four education packages (focussing on enabling the development of falls prevention strategies and self-efficacy) over the 13 weeks. Participants were invited to attend two one-to-one sessions: an initial assessment and goal setting session at local NHS/ university physiotherapy facilities, and a home visit to explain and demonstrate use of the online resources, support the home exercise programme and to problem solve any issues. Ongoing support was provided by online resources, a paper-based manual, bi-weekly reviews of participants’ online exercise logs by therapy staff and three, 2-h group sessions at local NHS/university physiotherapy facilities for peer support, group exercise and interactive learning activities over the course of the programme.

**Fig. 2:**
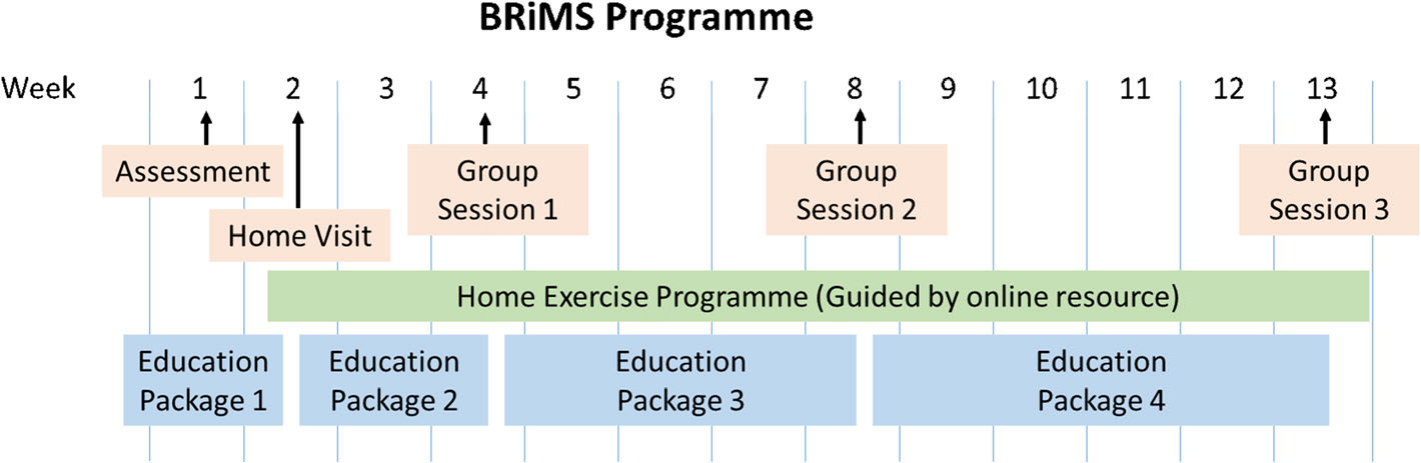
BRiMS programme delivery plan. SAE: serious adverse reactions (as classified by the study principle investigator/ chief investigator (see study protocol for details) [9]. Only a sub-group of participants were invited to participate in the qualitative interview.** One participant did not complete baseline data for the EQ5D-5L potential primary outcome

Participants allocated to the usual care group continued to receive their usual clinical input. Although usual care varies across the country [12], it rarely involves regular ongoing physiotherapy intervention on either an individual or a group basis. With the exception of the study assessments, they were not asked to attend any additional visits or sessions. Data on the nature/frequency of usual care for all participants was captured via a resource use questionnaire at each assessment time-point.

### Sample size

The target sample size was 60 participants across four UK sites in two regions (40 in the South West of England and 20 in Ayrshire, Scotland) to be recruited over 6 months. This would allow estimation of the overall retention rate with precision (using a 95% confidence interval) of at least ± 13%, improving to a ± 10% precision if the 27-week follow-up rate was around 80% [13, 14]. Assuming a non-differential follow-up rate of 80%, this recruitment target was anticipated to provide follow-up data on a minimum of 24 participants in each of the allocated groups, sufficient to calculate estimates of variability for the proposed outcome measures to inform indicative sample size calculations for the definitive trial.

### Outcomes

Outcome measures appropriate to each of the study objectives are listed in Table 1, with further detail included in Additional file 1. Potential primary and secondary outcome measures were chosen based on their relevance to the aims of the BRiMS programme and that there was psychometric evidence to support their use. Although the reduction of accidental falls was a key aim of BRiMS, falls rates (calculated from prospectively completed falls diaries, returned every 2 weeks) were considered a potential secondary rather than primary outcome, due to concerns about the reliability and validity of self-report falls diary data [15]. Instead a psychometrically robust mobility measure was chosen as a potential primary outcome, on the basis that this was likely to reflect changes in “safe” mobility which was a key goal of the BRIMS programme”

**Table 1:**
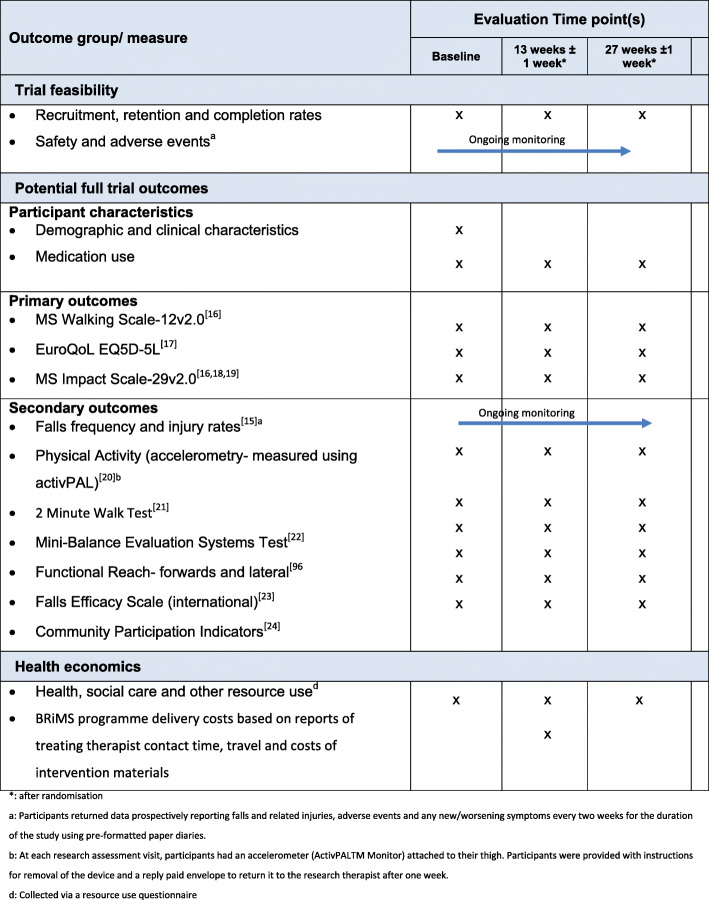
Outcome measures and data collection schedule [16–24]

*After randomisation

^a^Participants returned data prospectively reporting falls and related injuries, adverse events and any new/worsening symptoms every 2 weeks for the duration of the study using pre-formatted paper diaries

^b^At each research assessment visit, participants had an accelerometer (ActivPALTM Monitor) attached to their thigh. Participants were provided with instructions for removal of the device and a reply paid envelope to return it to the research therapist after 1 week

^d^ Collected via a resource use questionnaire

### Progression criteria

A number of progression criteria were pre-defined in discussion with the Trial Steering Committee (TSC):

1. A minimum of 80% recruitment within the planned 6-month recruitment window
2. A minimum of 80% participants randomised to BRiMS engaging with the programme (defined as attending the initial face-to-face clinic visit and home visit).
3. A minimum of 80% completion rate of at least one of the proposed primary outcome measures amongst participants attending the planned primary end-point of 27 (± 1 week).
4. That the total resource estimated to conduct the definitive trial is within a level that is likely to attract funding.

### Data analyses

A detailed statistical analysis plan was approved by the TSC prior to database lock. The statistical analyses were undertaken using StataSE version 14, supplemented where required by R [25].

Analyses were undertaken on a modified Intention To Treat (mITT) basis, with additional analysis of the falls data as outlined below, and in accordance with guidelines for pilot and feasibility trials [26]. Descriptive statistics were used to summarise patient eligibility, recruitment, allocation and retention, demographic and clinical characteristics, outcome measures and their completeness. Where appropriate, parameter estimates (e.g. between-group differences, both unadjusted and adjusted for baseline values where available) are presented with confidence intervals but no formal hypothesis testing was undertaken [26]. Outliers were identified and reported but not removed from the descriptive statistics unless otherwise stated. For the validated patient reported outcome measures, MS Walking Scale-12v2.0 [16] (MSWS-12v2), MS Impact Scale-29v2.0 [16, 18, 19] (MSIS-29v2) and Falls Efficacy Scale (international) [23] (FESi), established methods for imputing missing item-level data were implemented when the minimum requirements were met [27–29]. A validated imputation method was not available for the Community Participation Indicators [24] score and so summaries for this score are based on complete data only.

Rates of falls and injurious falls were calculated per person per year, using two different methods: (a) the “mITT” analysis assumed that if a participant did not complete or return a diary entry for a particular day, they did not fall (i.e. missing values were replaced with zeroes/no fall); (b) the “Observed” analysis used only the completed diary data. The rates were compared between allocated groups using unadjusted rate ratios (intervention: usual care), with bootstrapped confidence intervals.

Mean health state values and quality adjusted life-years (QALY) estimates used in the health economics analysis were based on the EQ5D-3L [30] derived from the EQ-5D-5L health states and the MS-specific preference based measure, the MSIS-8D [31] (derived from participant reports for the MSIS-29) collected throughout the study. The QALY combines length and quality-of-life in a single outcome measure. Each year of life is weighted by quality-of-life during that time. Quality-of-life is represented by QALY weights on a scale from zero (equivalent to being dead) to one (perfect health). QALY weights can also be negative, representing quality-of-life thought worse than being dead. A higher number of QALYs indicates a better health outcome. NICE advice is to use the EQ5D-3L rather than the EQ5D-5L [32]; therefore, the EQ5D-5L was mapped to the EQ5D-3L using the “cross walk” technique [30].

## Results

### Feasibility outcomes

#### Recruitment, randomisation, retention and engagement (Fig. 3)

##### Recruitment

Of the 232 subjects screened over 5 months, 44 specifically declined and a further 20 were deemed ineligible on screening, leaving 56 consented participants (satisfying progression criterion 1). The main reasons for individuals declining to participate were the time commitment (*n* = 16/44, 36%), and lack a computer or tablet access /poor IT literacy (*n* = 14/44, 32%). Despite using a range of recruitment procedures, thirty-seven (66%) of the 56 consented participants were recruited via personal approach by research support staff or local clinicians.

**Fig. 3:**
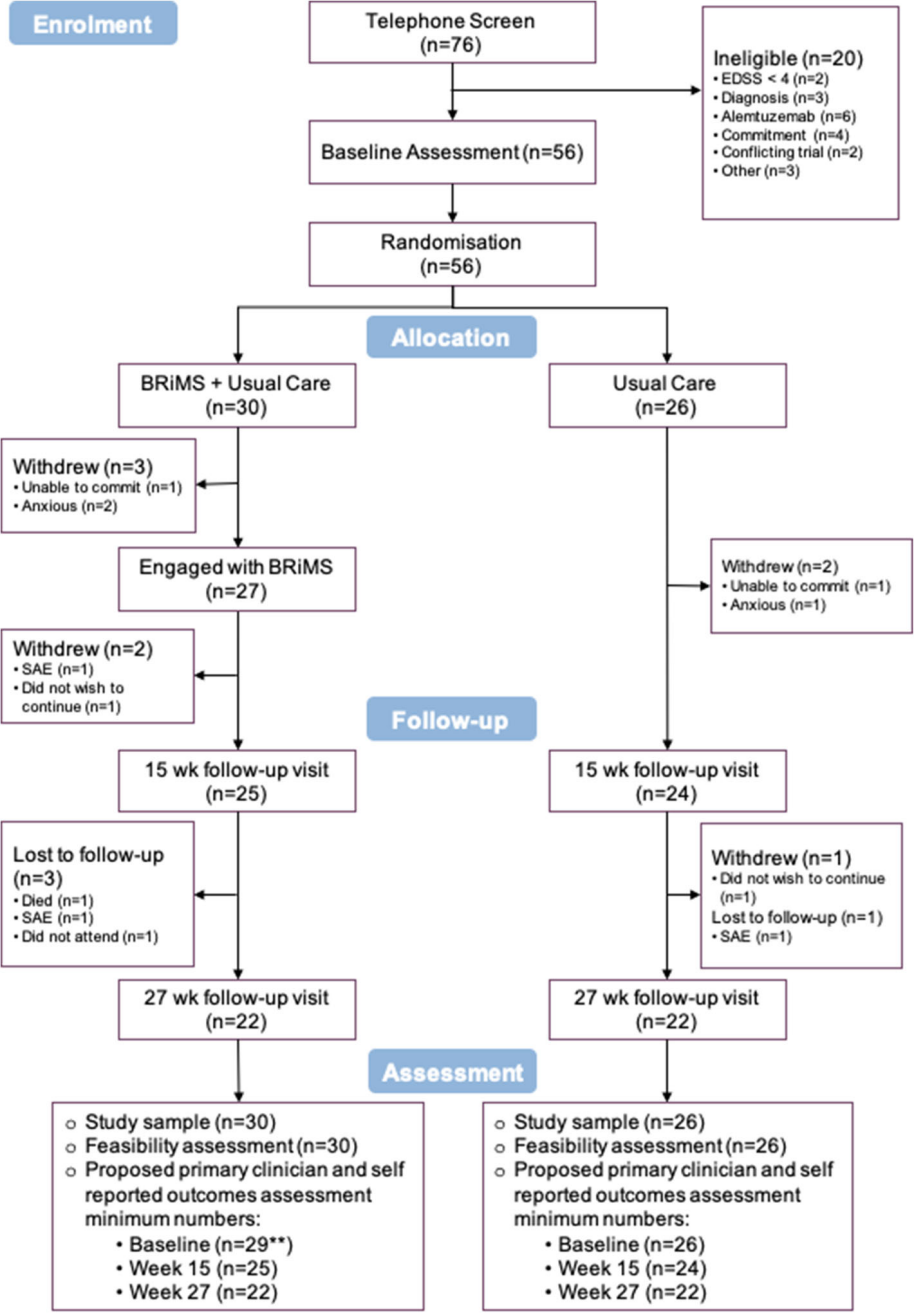
CONSORT flow diagram

##### Randomisation

Randomisation procedures were implemented successfully, resulting in the allocation of 30 participants to the BRiMS plus usual care group, and 26 to the usual care group.

##### Retention

The overall retention rate at week 27 was 79% (95% CI 66 to 88%); there was a higher withdrawal rate in the intervention group.

##### Programme engagement

Twenty-seven of the 30 participants (90%) allocated to the intervention group met the criteria for engagement with the programme (satisfying progression criterion 2).

##### Safety and serious adverse events (SAEs)

There were nine reports of SAEs from seven individuals over the study period (two usual care, five intervention). The two individuals who each reported two SAEs were in the intervention group. Despite participants all being classified with SPMS, four of the SAEs were reported to relate to MS relapses, the others to unrelated medical problems (*n* = 1) or falls with injuries requiring hospitalisation (*n* = 3; 2 intervention, 1 usual care). No SAE was assessed to be related to the BRiMS intervention.

### Trial outcome objectives

#### Outcome measure completion rates

At each assessment, the completion rate of each outcome measure was in excess of 98% (satisfying progresssion criterion 3). However, the overall return rate for the patient-reported falls diary was 62%. There was also incomplete recording within the diaries that were returned, meaning that data were available for 58% of the expected total number of days for falls, and 41% of the expected total for injurious falls. The diary return rate was different between allocated groups (54% BRiMS, 78% usual care).

#### Demographic and clinical characteristics at baseline

Table 2 details summary statistics of the participants’ baseline characteristics by allocated group and for the total sample: For full details, see final study report [11]. The groups were broadly comparable at baseline, although there were differences in disease severity (EDSS), anxiety/depression and cognition (symbol digit modalities test).

**Table 2:** Summary statistics of participants’ clinical and demographic data at baseline

Number of participants (%)	Usual care ***N*** = 26	BRiMS ***N*** = 30	Total ***N*** = 56
**Age (years)**			
Mean (SD)	60.0 (8.5)	58.7 (10.8)	59.3 (9.7)
[Min–max]	[46.0–81.0]	[34.0–80.0]	[34.0–81.0]
**Gender**			
Male	9 (34.6)	10 (33.3)	19 (33.9)
Female	17 (65.4)	20 (66.7)	37 (66.1)
**Living arrangements**^c^			
Alone	9 (34.6)	7 (23.3)	16 (28.6)
Spouse/partner	15 (57.7)	19 (63.3)	34 (60.7)
Parent/s	1 (3.8)	2 (6.7)	3 (5.4)
Child/ren	4 (15.4)	4 (13.3)	8 (14.3)
Other		1 (3.3)	1 (1.8)
**Occupation status**			
Unemployed	1 (3.8)	2 (6.7)	3 (5.4)
Part-time work	4 (15.4)	2 (6.7)	6 (10.7)
Full-time work	2 (7.7)	1 (3.3)	3 (5.4)
Age retired	5 (19.2)	5 (16.7)	10 (17.9)
Medically retired	14 (53.8)	19 (63.3)	33 (58.9)
**EDSS**^**b**^			
Median (LQ-UQ)	6.0 (6.0–6.5)	6.5 (6.0–6.5)	6.3 (6.0–6.5)
[Min-max]	[4.0–7.0]	[6.0–7.0]	[4.0–7.0]
**Cognition: SDMT**^**c**^			
Mean (SD) [Min–max]	44.5 (15.1) [7.0–77.0]	39.1 (10.9) [20.0–60.0]	41.6 (13.2) [7.0–77.0]
**Incontinence (previous 4 weeks)**			
Not at all	14 (53.8)	13 (43.3)	27 (48.2)
Once	1 (3.8)	3 (10)	4 (7.1)
2 to 4 times	3 (11.5)	7 (23.3)	10 (17.9)
>Weekly	5 (19.2)	3 (10)	8 (14.3)
Daily	3 (11.5)	4 (13.3)	7 (12.5)
**Three-month fall history**			
Not fallen^a^		1 (3.3)	1 (1.8)
Twice	7 (26.9)	5 (16.7)	12 (21.4)
3–5 times	11 (42.3)	13 (43.3)	24 (42.9)
More often	8 (30.8)	11 (36.7)	19 (33.9)
**Indoor walking aids**^c^			
1 stick/crutch	9 (34.6)	13 (43.3)	22 (39.3)
2 sticks/crutches	5 (19.2)	4 (13.3)	9 (16.1)
Walker/frame	8 (30.8)	12 (40)	20 (35.7)
Wheelchair	4 (15.4)	4 (13.3)	8 (14.3)
**Outdoor walking aids**^c^			
1 stick/crutch	17 (65.4)	18 (60)	35 (62.5)
2 sticks/crutches	10 (38.5)	7 (23.3)	17 (30.4)
Walker/frame	9 (34.6)	14 (46.7)	23 (41.1)
Wheelchair	12 (46.2)	15 (50)	27 (48.2)
**Number of medications**			
Median (LQ–UQ)	4 (2–7)	5 (2–7)	4 (2–7)
[Min–Max]	[0–10]	[0–17]	[0–17]
**Current co-morbidities**^c^			
COPD/asthma	1 (3.8)	2 (6.7)	3 (5.4)
Coronary heart disease/hypertension	1 (3.8)	1 (3.3)	2 (3.6)
Depression/anxiety	4 (15.4)	7 (23.3)	11 (19.6)
Diabetes	1 (3.8)	1 (3.3)	2 (3.6)
Migraine	3 (11.5)	1 (3.3)	4 (7.1)
Osteoarthritis	3 (11.5)	6 (20)	9 (16.1)
Osteoporosis	5 (19.2)	2 (6.7)	7 (12.5)
Other	10 (38.5)	11 (36.7)	21 (37.5)
Other neurological condition	1 (3.8)	1 (3.3)	2 (3.6)

^a^There was no option for participants to report falling once

^b^Expanded disability status scale; *SDMT* symbol digit modalities test

^c^ Participants could enter in multiple options; therefore percentages may not add up to 100

#### Outcomes

Table 3 shows summary statistics together with the simple between-group mean differences, mean differences after adjustment for corresponding baseline score, and the indicative minimal clinically important difference (MCID), where available, for the proposed primary outcome measures. Baseline scores indicate significant mobility impairments and falls-related concern. On average, participants in the intervention group scored worse than those in the usual care group at baseline. The adjusted mean differences in the MSWS-12v2 and the MSIS-29v2 indicate that the BRiMS group improved more at 15 weeks relative to the usual care group; the adjusted between-group difference for MSWS-12v2 (physical) exceeded the MCID [21] at both 15 and 27 weeks.

**Table 3:** Summary statistics, mean, standard deviation (SD) and range, and between-group mean differences of the potential primary outcome measures

	Time point	Usual care		BRiMS		Difference between allocated groups (BRiMS–usual care) Mean (95% CI)		Minimal clinically important difference (MCID), where available
		N	Mean (SD) [Min–max]	N	Mean (SD) [Min–max]	Unadjusted	Adjusted	
**MSWS-12v2**^**a**^ **(range 0–100)**	**Baseline**	26	79.6 (14.4)	30	84.2 (16.2)			Between 4.0 and 6.0 [33]
			[52.0–100.0]		[45.0–100.0]			
	**Week 15**	24	79.8 (13.9)	25	75.6 (19.4)	− 4.2 (− 14 to 5.5)	− 10.6 (− 18.9 to 2.2)	
			[48.0–100.0]		[33.0–100.0]			
	**Week 27**	22	79.5 (21.9)	22	75.4 (16.8)	− 4.0 (− 15.9 to 7.8)	− 7.7 (− 17.2 to 1.8)	
			[21.0–100.0]		[40.0–100.0]			
**EQ5D-3L**^**b**^ **(crosswalk)**	**Baseline**	26	0.58 (0.16)	29	0.54 (0.17)			0.05–0.08 [34]
			[0.04–0.77]		[− 0.04–0.88]			
	**Week 15**	24	0.60 (0.18)	25	0.59 (0.17)	− 0.01 (− 0.11 to 0.09)	0.03 (− 0.07 to 0.14)	
			[0.20–0.91]		[− 0.01–0.88]			
	**Week 27**	22	0.59 (0.25)	22	0.57 (0.11)	− 0.02 (− 0.13 to 0.10)	0.02 (− 0.09 to 0.14)	
			[− 0.13–0.91]		[0.30–0.77]			
**MSIS-29v2**^**a**^ **(physical)** **(range 0–100)**	**Baseline**	26	64.2 (21.7)	30	64.8 (16.4)			8.0 [35]
			[25.0–97.0]		[32.0–93.0]			
	**Week 15**	24	59.4 (23)	25	54.8 (19.5)	− 4.6 (− 16.8 to 7.7)	− 4.9 (− 13.2 to 3.5)	
			[13.0–98]		[13–92]			
	**Week 27**	22	59.0 (24.9)	22	57.9 (15.2)	− 1.2 (− 13.7 to 11.4)	0.6 (− 7.8 to 9)	
			[0.0.–92]		[27–88]			
**MSIS-29v2**^**a**^ **(psychological)** **(range 0–100)**	**Baseline**	26	45.1 (29.7)	30	50.4 (22.8)			N/A
			[0.0–85]		[4.0–96]			
	**Week 15**	24	43.3 (26.8)	25	43.7 (19)	0.5 (− 12.8 to 13.8)	− 5.0 (− 15.5 to 5.5)	
			[0–89]		[0.0–70.0]			
	**Week 27**	22	40.0 (26.8)	22	43.3 (22.6)	3.3 (− 11.8 to 18.4)	− 0.4 (− 9.9 to 9)	
			[0.0–93]		[7.0–81]			

*MSWS* MS walking scale, *MSIS* MS impact scale

^a^Decrease in score indicates improvement

^b^Increase in score indicates improvement

Potential secondary outcomes are summarised in Additional file 2. The adjusted mean differences indicate that the BRiMS group improved more at 15 and 27 weeks relative to the usual care group in most proposed outcome measures; however, all adjusted between-group mean differences were smaller than established MCID (where available), with wide 95% confidence intervals.

*N/A* not available, *Unadjusted* the mean difference between the allocated groups (BRiMS-usual care) with 95% confidence interval for potential primary outcomes. *Adjusted* each participants’ baseline score was subtracted from their follow-up score, and we report the mean difference between the allocated groups (BRiMS-usual care) with 95% confidence interval for potential primary outcomes

#### Falls data

There was substantial variation between individual falls reports over the 27-week study period (range 0–459 falls, as verified through telephone contact with the participant). One participant accounted for over half the reported falls in the usual care group; therefore, this individual was classified as an outlier and removed from the falls diary analyses presented, leaving a total of 715 falls.

#### Falls rates (see Table 4)

The rates of falls and injurious falls were lower in the BRiMS than the usual care group; however, the confidence intervals were wide and all included the null value (one).

**Table 4:** Falls and injurious falls rates (per person per year)

	Observed		ITT	
	BRiMS (***N*** = 26)	Usual Care (***N*** = 21)	BRiMS (***N*** = 30)	Usual care (***N*** = 25)
**Falls (rate per person per year)**				
Rate	38.1	39.1	21.9	27.0
Rate ratio (95% CI)^a^	0.97 (0.40 to 2.22)		0.81 (0.41 to 2.26)	
**Injurious falls (rate per person per year)**				
Rate	3.8	7.1	2.2	4.9
Rate ratio (95% CI)^a^	0.53 (0.40 to 2.21)		0.44 (0.41 to 2.23)	

^a^Bootstrapped 95% confidence intervals (CI)

### Indicative sample sizes for the anticipated definitive trial

The sample size calculations were undertaken for the proposed primary outcome of MSWS-12v2 at the primary endpoint of 27 weeks, to detect an improvement of 5.2 units [33] at the two-sided, 5% significance level with 90% power. Sample sizes were also adjusted for loss to follow-up rate of 70%.

As the definitive trial would be an individually randomised group treatment trial [36], the analysis would use a multi-level modelling approach, including adjustment for the baseline MSWS-12v2 score and allowing for the partially clustered data. It is assumed that participants allocated to the intervention arm would be clustered within small groups (~ 5 participants), whilst participants allocated to the usual care arm would not be clustered. Therefore, the indicative sample size calculations account for a potential ‘group’ effect by incorporating the intra-cluster correlation (ICC) [36, 37]. Given that the intervention is standardised and that the number of BRiMS group-based sessions is small, it is assumed the ICC will be small. However to account for any potential clustering effect, the base case assumes a conservative ICC of 0.05.

In this feasibility study, the point estimate of the SD of MSWS12-v2 at 27 weeks was 19.4 units, with one-sided 80% upper bound of 21.5. However, a slightly inflated SD of MSWS12-v2 of 23 units is assumed, based on pooling estimates from previous relevant studies [38, 39].

Correlation estimates for MSWS12-v2 between baseline and follow-up in the SWIMS project were 0.85–0.89 [40], indicating an adjustment for this correlation should be included in the sample size calculation. The correlation in this study was 0.59, with one-sided 80% lower bound of 0.50. Therefore, the base case assumes a correlation of 0.6.

The sample size calculations were performed in STATA using the clsampsi [37] command. The base case indicated a recruitment target of 836 participants in order to follow up 584 participants. Additional file 3 shows indicative sample sizes under a range of assumptions and shows that the main drivers of the total sample size required are the standard deviation and the correlation between baseline and follow-up of MSWS-12v2 scores.

### Health economics analysis

Methods used for economic analysis proved practical and feasible. The health economics resource use questionnaires and therapist contact sheets had completion rates greater than 98% for all those who attended assessments.

The mean cost per person for delivery of the BRiMS programme was £323. This is based on data collected on staff time by type of contact and staff type (NHS Band 7), collected within-study, aligned with published unit cost data; a mean of one clinic visit, one home visit, 5.26 online contacts and three group contacts. See the full project report [11] for further detail.

Detailed data on resource use is included in Additional file 4. Participants reported relatively modest levels of resource use over the study period, mostly focussed around items of primary and secondary care. There was little medication use reported by participants, which aligned with the study inclusion criteria, and was consistent with expectations as all participants had progressive MS. Estimated medication costs were associated with one person in each group reporting use of disease modifying therapy over the 27-week follow-up (for 25 weeks in the usual care group, and 6 weeks in the intervention group).

There was consistent reporting of informal care provision, with the reported mean hours per week similar across groups (24–25 h per week), estimated at a weekly cost of approximately £445 per participant; this being a relatively large cost component, currently provided via unpaid informal care inputs. Data on time off work by friends/relatives to support the participant was also captured, with no reports in the BRiMS group, and one participant in the usual care group reporting 13 days (mean of 0.59 days/participant in the usual care group).

As reported in detail elsewhere [11], there was some redundancy in the questionnaire items (i.e. no or minimal reports of resource use), which suggests a potential to reduce the questionnaire length in a future definitive trial.

### Health state values (EQ-5D, MSIS-8D) and quality adjusted life-years (QALYs)

The data collection to inform assessment of health state values was effective, with low levels of data loss. Table 5 summarises the estimated health state values and QALY estimates.

**Table 5:** Health state values and QALYs

	Usual care					BRiMS				
	Mean	(SD)	Min	Max	***N***	Mean	(SD)	Min	Max	***N***
**Baseline data:**										
EQ5D-3L	0.58	(0.16)	0.04	0.77	26	0.54	(0.17)	− 0.04	0.88	29
EQ5D-5L	0.66	(0.20)	0.07	0.89	26	0.63	(0.17)	0.22	0.95	29
MSIS-8D	0.51	(0.21)	0.08	0.80	26	0.49	(0.15)	0.21	0.76	30
**Week 15 data:**										
EQ5D-3L	0.60	(0.18)	0.20	0.91	24	0.59	(0.17)	− 0.00	0.88	25
EQ5D-5L	0.69	(0.18)	0.19	0.95	24	0.67	(0.17)	0.26	0.95	25
MSIS-8D	0.54	(0.20)	0.13	0.82	24	0.56	(0.16)	0.22	0.83	25
**Week 27 data:**										
EQ5D-3L	0.59	(0.25)	− 0.13	0.91	22	0.57	(0.11)	0.30	0.77	22
EQ5D-5L	0.67	(0.25)	0.05	0.95	22	0.65	(0.15)	0.38	0.89	22
MSIS-8D	0.56	(0.19)	0.08	0.88	22	0.54	(0.17)	0.18	0.77	22
**Estimated QALYs (over 27 weeks):**										
EQ5D-3L	0.30	(0.08)	0.13	0.43	22	0.30	(0.05)	0.20	0.42	22
EQ5D-5L	0.34	(0.09)	0.11	0.46	22	0.34	(0.07)	0.22	0.47	22
MSIS-8D	0.28	(0.10)	0.09	0.42	22	0.29	(0.06)	0.20	0.40	22

## Discussion

The results from this feasibility study inform the design of a future definitive randomised controlled trial of this exercise and education programme to improve safe mobility and reduce falls in people with progressive MS.

### Feasibility

Our pre-specified thresholds for recruitment, retention and data collection were satisfied [9]. Significant variability was identified in recruitment rates depending on the approach used. Previous studies have emphasised the need for a multi-faceted recruitment strategy [41]; however, our results highlight the importance of a personal approach by clinicians or research staff.

Overall loss to follow-up was within the 20% anticipated. There was a discrepancy between retention rates in the two arms of the study, and in particular, the dropout rate in the intervention group was higher than anticipated. In comparison, a review of 26 exercise intervention studies reported combined dropout rates of 15% and 16% for intervention and usual care groups respectively [42]. We hypothesise that our higher dropout rate in the intervention group may be associated with expectations of the BRiMS programme. Further exploration of this aspect is required, supporting the notion that feasibility testing is only one stage in the cycle of developing complex interventions [43].

### Participant characteristics

In recognition of the prevalence of comorbidity in MS, our recruitment criteria set out to be as inclusive as possible, and the sample characteristics were in line with publications in this field [44]. Whilst the allocation between groups was similar for most MS-related characteristics, the differing distribution of some (such as EDSS, anxiety/depression and cognition) could potentially affect outcomes, as evidenced by differences in baseline measures between the groups (e.g. MSWS-12v2, MiniBEST). This suggests that randomisation in a future definitive trial may require stratification by these characteristics, and that potential subgroup analyses should be considered when developing an a priori statistical analysis plan. The baseline characteristics also highlight that our sample was more severely balance and mobility impaired in comparison to a number of other studies with similar sample EDSS levels [21, 45]. For example, Gijbels et al. [46] report mean walking distances of 104 metres in the two minute walking test in a sample of 21 people with an EDSS between 4.5–6.5. In comparison, on average, our sample walked around 53 m at baseline. In addition, our sample reported higher levels of concern (as measured by the FESi) than other MS populations of mixed MS subtypes, although this is perhaps not surprising given their falls history and progressive MS [47].

### Proposed primary outcome measure

A key aim was to obtain data to inform the selection of a primary outcome measure for the definitive trial. The major consequence of falling for the individual is increasing mobility impairment, activity curtailment and loss of confidence [8, 48, 49]. Therefore, based on the existing evidence base, together with the results from this feasibility study, we recommend that the primary outcome for a definitive trial is the MSWS-12v2 [16]. Whilst a direct measure of injurious falls would be our favoured option given its clinical importance, our hesitancy in recommending this outcome is the recognised issues with the validity and reliability of falls diary data [50, 51], as evidenced by the problems with data completeness and accuracy we also experienced. If these issues can be resolved, then injurious falls should be re-considered as a primary outcome in a definitive trial. However, it is recognised that this would likely require a significantly larger sample size.

### Health economics data

Methods for collection of data (costs, outcomes) for a future economic evaluation were feasible and few challenges were faced in relation to this. The results highlight the relatively modest resource use of ‘formal’ health and care resources by the study participants, and the high use of ‘informal’ care and support. Our study is unable to determine if this pattern is through necessity (e.g. due to lack of resources) or choice; however, the findings reflect the importance of collecting comprehensive resource use data capturing both formal and informal care and support. The estimated health state values and QALY estimates are lower when using the MSIS-8D, and further research is recommended to consider why this may be (for example, being linked to specific domains of health-related QoL that may not be covered fully by the EQ-5D). However, the MSIS-8D indicates potential to show differences between groups over time.

### Strengths and limitations

A key strength of this feasibility study is that it used robust methodology, with comprehensive step-by-step documentation and evaluation of our processes, decisions and outcomes. However, there were some limitations. Most notably, despite adhering to best-practice recommendations [15] and with previous high return rates using similar methods [5, 52], the low return rate of the self-report paper-based falls diaries means that our falls data must be interpreted with caution. Our results highlight the need to find a valid and reliable method of collecting these data before falls can be considered as a potential primary outcome. Further, our assumption that if participants did not return a fall diary they did not fall, errs on the side of underestimating falls. In addition, the participants were only followed up for 3 months, and hence operational issues (such as study retention) and clinical outcomes are unknown for a longer follow-up period.

## Conclusions and recommendations

This study assessed the feasibility of undertaking a definitive trial to compare BRiMS plus usual care to usual care alone in a sample of people with SPMS who reported themselves as falling. We have demonstrated the study procedures to be feasible. Retention, programme engagement and outcome completion rates were all sufficient to satisfy our a priori progression criteria. Challenges were experienced in some areas, such as the completion of daily self-report fall diaries. A future trial should consider alternative methods of collecting these data. Estimated sample sizes for a definitive trial with MSWS-12v2 as the primary outcome range from 575 to 990 participants.

## Supplementary Information

**Additional file 1.** Summary statistics, mean, standard deviation (SD) and range, and between-group differences of the potential secondary outcome measures [15, 17, 20, 23, 24, 27, 28, 53–63].

**Additional file 2.** Potential secondary outcome analyses [46, 56, 64–66].

**Additional file 3.** Indicative sample sizes for a definitive trial of BRiMS

**Additional file 4.** Health and social care/informal care costs [67].

## Data Availability

Individual participant data that underlie the results of this study will be made available (following de-identification) on a controlled access basis, subject to suitable data sharing agreements. Requests for data sharing should be made to the Chief Investigator (CI, Freeman) in the first instance. Requesters will be asked to complete an application form detailing specific requirements, rationale and proposed usage. Requests will be reviewed by the CI and study Sponsor who will consider the viability and suitability of the request and the credentials of the requester. Where access to requested data is granted, requesters will be asked to sign a data sharing agreement. Requested data will be made available, along with supporting documentation (e.g. data dictionary) on a secure server or via other secure data transfer method.
