# Supplementary material for: Balance Right in Multiple Sclerosis (BRiMS): a feasibility randomised controlled trial of a falls prevention programme
Source: Pilot Feasibility Stud. 2021 Jan 4;7:2. doi: 10.1186/s40814-020-00732-9 (PMC7780657; doi:10.1186/s40814-020-00732-9)
Supplement: Supplementary file 1 — Additional file 1. Summary statistics, mean, standard deviation (SD) and range, and between-group differences of the potential secondary outcome measures [15, 17, 20, 23, 24, 27, 28, 53–63]. [file 40814_2020_732_MOESM1_ESM.docx]

**Trial Feasibility**

Data from screening, recruitment and follow-up logs were used to generate realistic estimates of eligibility, recruitment, consent and follow-up rates.

Safety and adverse event data were collected from monitoring forms completed by treating and research therapists, plus any participant reported incidents included in self-report diary returns.

**Potential full trial primary and secondary outcomes**

The trial used standardised clinician-rated and patient self-reported clinical outcomes, which have demonstrated good reliability and validity in people with MS.

**1. Primary outcomes**

**Walking -** Multiple Sclerosis Walking Scale–12 item (MSWS-12) Version 2.0^[27]^.

**Health related quality of life** EuroQoL [EQ5D-5L]^[17]^ and the 29-item **Multiple Sclerosis Impact Scale** [MSIS-29] Version 2.0^[28]^, which have been specified for use in health economic analyses in MS studies^[53]^.

**2. Secondary outcomes**

**Falls Frequency and Injury Rates**

Falls were defined as: “an unexpected event in which you come to rest on the floor or ground or lower level”^[54]^. In line with best practice guidance, the number of falls, injurious falls and associated use of medical services were recorded prospectively using a patient completed daily diary returned to the CTU in a FREEPOST envelope on a fortnightly basis^[15]^.

**Activity Level** using an activity monitor (activPAL™, Paltechnologies Ltd, Glasgow)^[20]^.

**Walking Capacity** using the two-minute walk test (fastest speed) (2MWT). This has been recommended as the standard objective walking test to be used in MS interventional studies^[55]^.

**Balance** using the Mini-Balance Evaluation Systems Test (Mini-BEST)^[56]^ and the Functional Reach Test (forwards and lateral)^[57–59]^.

**Fear of Falling** using the 16-item self-report Falls Efficacy Scale (International) (FESi)^[23]^. This has been recommended as the standard objective measure of fear of falling by the Prevention of Falls Network Europe^[54]^.

**Community Integration** using the self-report Community Participation Indicators (CPI)^[24]^. This has been recommended as an objective measure of participation for use in falls prevention studies by the International MS Falls Prevention Research Network^[60]^.

**Health Economics**

Methods for the collection of resource use, cost, and outcome data were developed and tested in preparation for an economic evaluation alongside a full trial. Data on resource use associated with the set-up and delivery of the BRiMS intervention were collected via within trial reporting, including participant level contact and non-contact time for staffing input on delivery, equipment and consumable costs, training and supervision. Data on health and social care resource use were collected at participant level using a Participant Resource Use (RU) questionnaire, developed for this trial^[61]^. The EQ-5D-5L was used to estimate quality-adjusted life-years (QALYs), and is the expected primary economic endpoint (cost per QALY) in any future evaluation. The MSIS-8D^[62,63]^, an MS specific preference based (QALY) measure, was also used, as this is expected to be of value in future sensitivity analyses.
