# Supplementary material for: Balance Right in Multiple Sclerosis (BRiMS): a feasibility randomised controlled trial of a falls prevention programme
Source: Pilot Feasibility Stud. 2021 Jan 4;7:2. doi: 10.1186/s40814-020-00732-9 (PMC7780657; doi:10.1186/s40814-020-00732-9)
Supplement: Supplementary file 2 — Additional file 2. Potential secondary outcome analyses [46, 56, 64–66]. [file 40814_2020_732_MOESM2_ESM.docx]

| **Potential secondary outcome measures** | | | | | | | | | | | | | |
| --- | --- | --- | --- | --- | --- | --- | --- | --- | --- | --- | --- | --- | --- |
| **Variable** | **Time point** | **Usual care** | | | **BRiMS** | | | | | **Difference between allocated groups**  **(BRiMS – usual care)**  **Mean (95% CI)** | | | **Minimal clinically important difference (MCID), where available** |
|  |  | **N** | **Mean (SD)**  **[Min - Max]** | | **N** | **Mean (SD)**  **[Min - Max]** | | | | **Unadjusted** | | **Adjusted** |  |
| **Clinician-rated measures** | | | | | | | | | | | | | |
| **2MWT^b^***  **(metres)** | **Baseline** | 26 | 52.7 (27.2) | | 30 | | 53 (32) | | |  | | | 12.2 to 14.7^[64]^ or 19.21 metres^[46]^ |
|  |  |  | [7 - 138.5] | |  |  | [9 - 133] | | |  |  |  |  |
|  | **Wk 15** | 24 | 51.9 (29.5) | | 24 | | 53.3 (27) | | | 1.4 (-15 to 17.9) | | 0.3 (-9.4 to 10) |  |
|  |  |  | [8.5 - 120] | |  |  | [13 - 101] | | |  |  |  |  |
|  | **Wk 27** | 21 | 56.3 (31.1) | | 20 | | 55 (27.4) | | | -1.3 (-19.9 to 17.2) | | -2.2 (-14.6 to 10.2) |  |
|  |  |  | [12 - 126.8] | |  |  | [16 - 103] | | |  |  |  |  |
| **MiniBEST^b^**  **(Scale range 0-28)** | **Baseline** | 26 | 12.2 (3.8) | | 29 | | 10.7 (5.2) | | |  | | | 3.5^[56]^ |
|  |  |  | [6 - 21] | |  |  | [2 - 20] | | |  |  |  |  |
|  | **Wk 15** | 24 | 12.5 (6.2) | | 24 | | 14.2 (6.2) | | | 1.7 (-1.9 to 5.3) | | 2.6 (-0.1 to 5.4) |  |
|  |  |  | [1 - 25] | |  |  | [4 - 27] | | |  |  |  |  |
|  | **Wk 27** | 21 | 13.6 (6) | | 20 | | 14.1 (5.7) | | | 0.5 (-3.2 to 4.2) | | 1.2 (-1.2 to 3.6) |  |
|  |  |  | [3 - 27] | |  |  | [3 - 22] | | |  |  |  |  |
| **Forward FRT^b^**  **(cm)** | **Baseline** | 26 | 16.6 (6.4) | | 30 | | 18.1 (7.9) | | |  | | | 6.35-6.79 (vestibular disorders)^[65]^ |
|  |  |  | [1 - 28.7] | |  |  | [0 - 32.3] | | |  |  |  |  |
|  | **Wk 15** | 24 | 16.1 (6.4) | | 24 | | 20.8 (7.6) | | | 4.7 (0.6 to 8.8) | | 2.7 (-0.3 to 5.7) |  |
|  |  |  | [0 - 29.3] | |  |  | [7.7 - 38.7] | | |  |  |  |  |
|  | **Wk 27** | 21 | 17 (7.8) | | 20 | | 19.5 (5.9) | | | 2.5 (-1.9 to 6.9) | | 1 (-2.7 to 4.6) |  |
|  |  |  | [0 - 32] | |  |  | [6.3 - 33.7] | | |  |  |  |  |
| **Lateral FRT^b^**  **(cm)** | **Baseline** | 26 | 13.3 (6.2) | | 30 | | 13.4 (6.9) | | |  | | | N/A |
|  |  |  | [0 - 30] | |  |  | [0 - 27.3] | | |  | |  |  |
|  | **Wk 15** | 24 | 12.3 (5.2) | | 24 | | 16.7 (7.6) | | | 4.4 (0.6 to 8.2) | | 4.2 (0.9 to 7.5) |  |
|  |  |  | [0 - 20.7] | |  |  | [5 - 33.3] | | |  |  |  |  |
|  | **Wk 27** | 21 | 11.9 (5.4) | | 20 | | 16.1 (4.9) | | | 4.2 (0.9 to 7.5) | | 4 (1.1 to 6.9) |  |
|  |  |  | [0 - 21.3] | |  |  | [6 - 24.3] | | |  |  |  |  |
| **Self-reported outcomes** | | | | | | | | | | | | |  |
| **FESi^a^**  **(Scale range 16-64)** | **Baseline** | 26 | 43.7 (9.8) | | 30 | | 44.1 (9) | | |  | | | 8.2 (vestibular disorders)^[66]^ |
|  |  |  | [24.0 – 61.0] | |  |  | [27.0 – 60.0] | | |  |  |  |  |
|  | **Wk 15** | 24 | 44.3 (10) | | 25 | | 40.1 (8.1) | | | -4.2 (-9.4 to 1) | | -5.1 (-9.8 to -0.4) |  |
|  |  |  | [27.0 – 58.0] | |  |  | [28.0 – 55.0] | | |  |  |  |  |
|  | **Wk 27** | 22 | 44.5 (12) | | 22 | | 41.6 (8.4) | | | -2.9 (-9.2 to 3.4) | | -3.7 (-8.8 to 1.4) |  |
|  |  |  | [22.0 – 62.0] | |  |  | [24.0 – 55.0] | | |  |  |  |  |
| **CPI 1**  **(%)** | **Baseline** | 25 | 97.4 (39.2) | | 27 | | 101 (55.5) | | |  | | | N/A |
|  |  |  | [11.1 - 212.5] | |  |  | [38.9 - 266.7] | | |  |  |  |  |
|  | **Wk 15** | 24 | 107.5 (59.1) | | 24 | | 100.2 (38.7) | | | -7.3 (-36.3 to 21.7) | | 0.3 (-29.3 to 29.8) |  |
|  |  |  | [33.3 - 271.4] | |  |  | [38.9 - 187.5] | | |  |  |  |  |
|  | **Wk 27** | 22 | 108.3 (47.6) | | 22 | | 92.6 (34.6) | | | -15.8 (-41.1 to 9.5) | | -12.2 (-39 to 14.6) |  |
|  |  |  | [17.6 - 200] | |  |  | [41.2 - 146.2] | | |  |  |  |  |
| **CPI 2^b^**  **(Scale range 0-100)** | **Baseline** | 26 | 42.9 (8.3) | | 27 | | 40.8 (7.9) | | |  | | | N/A |
|  |  |  | [26.3 - 64.4] | |  |  | [26.3 - 53.1] | | |  |  |  |  |
|  | **Wk 15** | 24 | 40.8 (9.3) | | 24 | | 42.3 (9.9) | | | 1.5 (-4.1 to 7.1) | | 2 (-2 to 6.1) |  |
|  |  |  | [21.5 - 63.3] | |  |  | [11.4 - 57.3] | | |  |  |  |  |
|  | **Wk 27** | 22 | 41.7 (11.3) | | 22 | | 42.2 (9.4) | | | 0.5 (-5.8 to 6.8) | | 1.8 (-2.6 to 6.1) |  |
|  |  |  | [11.4 - 57.3] | |  |  | [17.8 - 54.7] | | |  |  |  |  |
| **CPI 3^b^**  **(Scale range 0-100)** | **Baseline** | 26 | 57.5 (10.4) | | 26 | | 53.7 (9.3) | | |  | | | N/A |
|  |  |  | [38.1 - 81.4] | |  |  | [39 - 81.4] | | |  |  |  |  |
|  | **Wk 15** | 24 | 58.4 (14) | | 24 | | 54.1 (11.1) | | | -4.2 (-11.6 to 3.1) | | -2.1 (-6.5 to 2.4) |  |
|  |  |  | [39 - 100] | |  |  | [37.1 - 88.2] | | |  |  |  |  |
|  | **Wk 27** | 22 | 59.4 (15.4) | | 22 | | 54.2 (10.5) | | | -5.2 (-13.2 to 2.8) | | -0.4 (-5.3 to 4.4) |  |
|  |  |  | [23.7 - 88.2] | |  |  | [36.2 - 77.3] | | |  |  |  |  |
| **Accelerometry data (measured by ActivPAL^TM^)** | | | | | | | | | | | | | |
| **Step count** | Baseline | 26 | 3286.4 (2760.7) | 28 | | | | 2654.9 (1861.1) |  | | | |  |
|  |  |  | [256.3 - 11756.4] |  |  |  |  | [11.7 - 7543.2] |  |  |  |  |  |
|  | Wk 15 | 23 | 3443.6 (3721.8) | 23 | | | | 2841.4 (1841) | -602.2 (-2347.1 to 1142.7) | | -165.7 (-1047.6 to 716.2) | | N/A |
|  |  |  | [283.2 - 13034.5] |  |  |  |  | [5.2 - 7671.2] |  |  |  |  |  |
|  | Wk 27 | 22 | 2982.6 (3248) | 20 | | | | 3633.1 (2268.3) | 650.5 (-1113.7 to 2414.7) | | 699.8 (-495.3 to 1894.8) | | N/A |
|  |  |  | [166.7 - 14739.6] |  |  |  |  | [118.3 - 9027.2] |  |  |  |  |  |
| **Sitting/ Lying time**  **(hours)** | Baseline | 26 | 19.7 (1.7) | 28 | | | | 19.9 (2.1) |  | | | |  |
|  |  |  | [16.8 - 22.9] |  |  |  |  | [15.3 - 23.2] |  |  |  |  |  |
|  | Wk 15 | 23 | 19.8 (2.3) | 23 | | | | 19.7 (1.9) | 0.0 (-1.3 to 1.2) | | 0.3 (-0.8 to 1.4) | | N/A |
|  |  |  | [13.5 - 23.4] |  |  |  |  | [16.5 - 22.9] |  |  |  |  |  |
|  | Wk 27 | 22 | 19.7 (2.2) | 20 | | | | 19.0 (1.9) | -0.6 (-1.9 to 0.7) | | -0.2 (-1.1 to 0.8) | | N/A |
|  |  |  | [13.5 - 23.1] |  |  |  |  | [16.1 - 22.7] |  |  |  |  |  |
| **Standing time**  **(Hours)** | Baseline | 26 | 3.4 (1.4) | 28 | | | | 3.4 (1.9) |  | | | |  |
|  |  |  | [1.0 - 6.1] |  |  |  |  | [0.6 - 8] |  |  |  |  |  |
|  | Wk 15 | 23 | 3.3 (1.8) | 23 | | | | 3.5 (1.7) | 0.2 (-0.8 to 1.2) | | -0.3 (-1.2 to 0.7) | | N/A |
|  |  |  | [0.4 - 7.5] |  |  |  |  | [1.0 - 6.7] |  |  |  |  |  |
|  | Wk 27 | 22 | 3.5 (1.7) | 20 | | | | 4.0 (1.7) | 0.5 (-0.6 to 1.5) | | 0.0 (-0.8 to 0.8) | | N/A |
|  |  |  | [0.6 - 7.6] |  |  |  |  | [1.2 - 7.2] |  |  |  |  |  |
| **Stepping time**  **(Hours)** | Baseline | 26 | 0.9 (0.6) | 28 | | | | 0.8 (0.5) |  | | | |  |
|  |  |  | [0.1 - 2.9] |  |  |  |  | [0.0 - 2] |  |  |  |  |  |
|  | Wk 15 | 23 | 0.9 (0.9) | 23 | | | | 0.8 (0.5) | 0.5 (-0.6 to 1.5) | | 0.0 (-0.2 to 0.2) | | N/A |
|  |  |  | [0.1 - 3] |  |  |  |  | [0.0 - 1.9] |  |  |  |  |  |
|  | Wk 27 | 22 | 0.8 (0.7) | 20 | | | | 1.0 (0.5) | 0.2 (-0.2 to 0.5) | | 0.2 (-0.1 to 0.4) | | N/A |
|  |  |  | [0.1 – 3.0] |  |  |  |  | [0.1 - 2.2] |  |  |  |  |  |
| **Sit to stand**  **(Transitions)** | Baseline | 26 | 48.2 (16.9) | 28 | | | | 48.1 (19.8) |  | | | |  |
|  |  |  | [15.8 - 84.8] |  |  |  |  | [17.0 - 95.2] |  |  |  |  |  |
|  | Wk 15 | 23 | 47.0 (20.8) | 23 | | | | 51.6 (21.6) | 4.6 (-8 to 17.2) | | 2.0 (-4.6 to 8.6) | | N/A |
|  |  |  | [18.8 - 101.5] |  |  |  |  | [17.2 - 101] |  |  |  |  |  |
|  | Wk 27 | 22 | 45.0 (19.8) | 20 | | | | 56.2 (20.2) | 11.1 (-1.4 to 23.6) | | 4.7 (-4.1 to 13.6) | | N/A |
|  |  |  | [17.3 - 84.8] |  |  |  |  | [15.0 - 101.5] |  |  |  |  |  |

^2MWT: two minute timed walk test; MiniBEST: mini balance evaluation systems test; FRT: functional reach test; FESi: Falls efficacy scale (international); CPI: community participation indicators; *Results for the 2MWT do not account for sub 2 minute walk times or assistive devices. a: decrease in score indicates improvement; b: increase in score indicates improvement; N/A: not available; Unadjusted = the mean difference between the allocated groups (BRiMS - usual care) with 95% confidence interval for potential primary outcomes. Adjusted = each participants’ baseline score was subtracted from their follow-up score and we report the mean difference between the allocated groups (BRiMS - usual care) with 95% confidence interval for potential primary outcomes^
