# Supplementary material for: Balance Right in Multiple Sclerosis (BRiMS): a feasibility randomised controlled trial of a falls prevention programme
Source: Pilot Feasibility Stud. 2021 Jan 4;7:2. doi: 10.1186/s40814-020-00732-9 (PMC7780657; doi:10.1186/s40814-020-00732-9)
Supplement: Supplementary file 3 — Additional file 3. Indicative sample sizes for a definitive trial of BRiMS [file 40814_2020_732_MOESM3_ESM.docx]

|  | **Between-group difference at 6 months** | **Standard deviation** | **Cluster Size** | **ICC for intervention group** | **Unadjusted total sample size required to be followed-up** | **Total sample size required to be recruited - adjusted for 30% LTF** | **Correlation between baseline and 6 months' MSWS-12v2** | **Total sample Size required to be followed-up - adjusted for correlation** | **Total sample size required to be recruited - adjusted for correlation and 30% LTF** |
| --- | --- | --- | --- | --- | --- | --- | --- | --- | --- |
| **Base case** | 5.2 | 23 | 5 | 0.05 | 912 | 1303 | 0.6 | 584 | 836 |
|  | | | | | | | | | |
| **Vary ICC** | 5.2 | 23 | 5 | 0.075 | 954 | 1363 | 0.6 | 611 | 876 |
|  | 5.2 | 23 | 5 | 0.100 | 995 | 1421 | 0.6 | 637 | 914 |
|  | 5.2 | 23 | 5 | 0.125 | 1040 | 1486 | 0.6 | 666 | 951 |
|  | | | | | | | | | |
| **Vary standard deviation** | 5.2 | 19 | 5 | 0.05 | 625 | 893 | 0.6 | 400 | 576 |
|  | 5.2 | 21 | 5 | 0.05 | 761 | 1087 | 0.6 | 487 | 697 |
|  | 5.2 | 25 | 5 | 0.05 | 1080 | 1543 | 0.6 | 691 | 987 |
|  | | | | | | | | | |
| **Vary correlation** | 5.2 | 23 | 5 | 0.05 | 912 | 1303 | 0.5 | 684 | 979 |
|  | 5.2 | 23 | 5 | 0.05 | 912 | 1303 | 0.7 | 465 | 666 |
|  | | | | | | | | | |
| **Vary cluster size** | 5.2 | 23 | 6 | 0.05 | 936 | 1337 | 0.6 | 599 | 856 |
|  | 5.2 | 23 | 7 | 0.05 | 956 | 1366 | 0.6 | 612 | 878 |

^LTF: Loss to follow-up^
