# Supplementary material for: Balance Right in Multiple Sclerosis (BRiMS): a feasibility randomised controlled trial of a falls prevention programme
Source: Pilot Feasibility Stud. 2021 Jan 4;7:2. doi: 10.1186/s40814-020-00732-9 (PMC7780657; doi:10.1186/s40814-020-00732-9)
Supplement: Supplementary file 4 — Additional file 4. Health and social care/informal care costs [67]. [file 40814_2020_732_MOESM4_ESM.docx]

**Additional file 4: Health and social care/ informal care costs**

|  | **Usual Care** | | | **BRiMS** | | |
| --- | --- | --- | --- | --- | --- | --- |
|  | **Mean £** | **(SD)** | **N** | **Mean £** | **(SD)** | **N** |
| **Summary: Health and social care costs over 27 weeks** | | | | | | |
| Total Primary care | 213 | (224) | 22 | 190 | (162) | 22 |
| Total Secondary care | 349 | (249) | 22 | 1451 | (4,335) | 22 |
| Total Social and community care | 79 | (309) | 22 | 282 | (986) | 22 |
| **Total health and social care** | **640** | **(580)** | **22** | **1,922** | **(5,340)** | **22** |
| Total Medications | 261 | (921) | 22 | 45 | (209) | 22 |
| **Total All healthcare** | **902** | **(1,105)** | **22** | **1,967** | **(5,341)** | **22** |
| **Summary: Informal care cost (weekly) and cost for days off work over 27 weeks** | | | | | | |
| Total reported informal care (hrs/week) | 24.67 | (24.5) | 22 | 24.74 | (21.5) | 22 |
| Total cost of weekly informal care £^a^ | 444.14 | (441.27) | 22 | 445.30 | (387.59) | 22 |
| Friend/relative days off work (mean days/ participant) | 0.59 | (2.77) | 22 | 0.00 | (0.00) | 22 |
| Friend/relative days off work (mean cost/participant) | 72.28 | (339.02) | 22 | 0.00 | (0.00) | 22 |

^Here we use a unit cost (shadow price) for informal care of £18/hour, Curtis et al, 2016, p160[67]); a Mean costs calculated over the 27 week period: £11,992 (Usual Care), £12023 (BRiMS)^
